# Sleep in COPD: an understudied and clinically important issue

**DOI:** 10.48101/ujms.v131.14699

**Published:** 2026-11-03

**Authors:** Jenny Theorell-Haglöw

**Affiliations:** Department of Medical Sciences, Respiratory, Allergy and Sleep Research, Uppsala University, Uppsala, Sweden

**Keywords:** Chronic obstructive pulmonary disease (COPD), Sleep quality, Sleep disturbances, Sleep disorders

## Abstract

This review provides an overview of objective and subjective sleep characteristics in the general chronic obstructive pulmonary disease (COPD) population and discusses associations with airway symptoms, psychological factors, quality of life, and common sleep disorders. Available studies indicate that patients with COPD often exhibit altered sleep architecture, including increased light sleep, reduced slow-wave and rapid eye movement (REM) sleep, frequent arousals, reduced sleep efficiency, and shorter total sleep duration. Subjectively, 40–70% of patients report poor sleep quality, characterized by difficulty initiating or maintaining sleep and increased daytime sleepiness. Nocturnal respiratory symptoms such as dyspnea, cough, wheeze, sputum production, and hypoxemia may contribute to sleep disruption. Common sleep disorders such as insomnia, obstructive sleep apnea (OSA), and restless legs syndrome (RLS) are seen more frequently in COPD, and poor sleep may also negatively affect COPD outcomes through mechanisms such as systemic inflammation, reduced physical activity, and impaired treatment adherence. Sleep disturbances are further associated with anxiety, depression, fatigue, and reduced quality of life. Assessing sleep and sleep quality and treating both COPD and sleep disorders effectively in this patient population are of importance and would likely improve not only sleep and airway symptoms but also quality of life and overall clinical outcomes.

## Introduction

Sleep is a biological function (1) essential for physical health, cognitive functioning, and emotional regulation (2). Adequate sleep supports energy conservation, tissue restoration, and neural plasticity, (3) whereas chronic sleep deprivation is associated with increased risk of mood disturbances, impaired daytime function, and cardiometabolic disorders (2).

Sleep in chronic obstructive pulmonary disease (COPD) has often been studied from the treatment perspective, assessing impact on sleep quality from positive airway pressure (PAP) for hypercapnic respiratory failure (4) or the effect of pulmonary rehabilitation on sleep quality (5). This review’s overarching aim is to provide an overview of objectively and subjectively measured sleep in the general COPD patient population and also give some perspectives on sleep and COPD with focus on airway symptoms and other outcomes, as well as associations with psychological factors and quality of life. In addition, the prevalence of common sleep disorders in COPD is covered. Together, this strives to highlight the importance of sleep in respiratory disease.

## Assessing sleep and sleep quality

### Sleep duration

Sleep duration can be assessed both objectively through polysomnography (PSG) or actigraphy and subjectively through questionnaires. PSG is considered gold standard when assessing sleep and in investigating sleep disorders. PSG includes electroencephalography, electrooculography, and chin electromyography for sleep staging. In addition, airflow, respiratory effort measures, pulse oximetry, and body position assess breathing disorders. Furthermore, electrocardiography is assessed, and leg electromyography assesses limb movements during sleep. Together, these not only provide the basis for scoring sleep and sleep measures such as total sleep time, different sleep stages, sleep efficiency, number of awakenings, wake time within total sleep period (WTSP) but also measure sleep-disordered breathing (including OSA) and limb movements during sleep.

Another way to objectively assess sleep is using actigraphy. Actigraphy devices are portable sleep devices that can be worn comfortably over extended periods without interfering with the participants’ daily routine. They have been shown to give good estimates of total sleep time as compared to the gold standard of PSG for assessing sleep duration (6).

Typically, questionnaires to assess sleep duration assess habitual sleep duration, sometimes by weekday and weekend sleep. In addition, information on sleep latency may also be included.

### Sleep quality

There is no objective measure to assess sleep quality. However, in previous studies, perceived poor sleep quality has been shown to be related to short total sleep time, long WTSP, low sleep efficiency, and a high number of awakenings (7). Nonetheless, studies also indicate that rather than objective sleep variables, subjective fatigue is central in the perception of poor sleep (8).

Sleep quality is most commonly assessed via questionnaires, the most commonly used being the Pittsburgh Sleep Quality Index (PSQI) (9). The PSQI comprises 19 questions and assesses subjective sleep quality, sleep latency, sleep duration, habitual sleep efficiency, sleep disturbances (including insomnia symptoms, nocturia, nocturnal cough and snoring), use of sleeping medication, and daytime dysfunction over the past month. A global score of the including components is generated, and a global PSQI score ≥5 is indicative of poor sleep quality (9). Within the commonly used COPD assessment tool (CAT), there is one question on sleep where patients score their sleep on a 5-point Likert scale from *0=I sleep soundly* to *5=I do not sleep soundly because of my lung condition* (10).

## Objectively measured sleep in COPD

Studies on objectively measured sleep in COPD are still few; nonetheless, studies using PSG indicate a higher prevalence of light nonrapid eye movement (REM) sleep stages and frequent arousals, more sleep fragmentation, diminished slow wave and REM sleep, shorter sleep duration, and more frequent arousals in patients compared with healthy controls (11–13). Also in a more recent COPD outpatient cohort study, Hynninen *et al.* indicate reduced sleep efficiency in COPD patients (14). We have previously shown that sex differences in objective sleep may occur, with men with COPD having longer recorded sleep latency than the male controls, a result not found in women (15). It is, however, important to remember that many studies using objective measures have primarily aimed to study effects on night-time saturation and not sleep architecture and also have not distinguished patients with and without sleep-disordered breathing (11, 12, 16, 17). Nonetheless, daytime hypoxemia has been shown to be independently associated with impaired sleep efficiency (13).

Also when using actigraphy, objective sleep has been shown to be disturbed in COPD patients, with more fragmentation in circadian rhythm (18), longer sleep latency, and reduced sleep duration (19).

## Sleep quality in COPD

Individuals with COPD frequently report sleep disturbances and symptoms of insomnia, including greater difficulty initiating and maintaining sleep as well as increased daytime sleepiness compared with the general population (11, 20). Poor sleep quality is particularly evident in patients with severe COPD when compared with age-matched normative populations. Overall, up to 70% of patients with COPD report difficulty sleeping, which is two to three times more frequent than in the general population (21–23). In addition, a higher prevalence of nightmares and daytime sleepiness has been reported among individuals with COPD (24). Furthermore, physiological mechanisms such as hypoventilation during sleep is often more pronounced in COPD due to factors such as airflow obstruction, lung hyperinflation, respiratory muscle dysfunction, and reduced ventilatory responses to hypercapnia and/or hypoxia, which may contribute to disturbed sleep (23). Adding to this, regular use of hypnotic medications is more commonly reported in this population (11, 25), and certain medications may also contribute to nocturnal hypoventilation (26), possibly worsening sleep.

Using machine learning from clinical data of COPD patients, Öz et al. showed that sleep quality in COPD could be predicted (27). High annual exacerbation and hospitalization rates, the presence of daytime sleepiness and cough symptoms, active smoking, and regular use of inhaled corticosteroids, as well as high CO_2_ levels, negatively affect sleep quality (27).

## Sleep and COPD: relationship with airway symptoms and other outcomes

Airway symptoms during the night are common in COPD and can substantially affect sleep. Night-time dyspnea is prevalent in COPD and may disrupt sleep, potentially leading to tiredness and impaired daytime functioning (28). In addition, symptoms such as cough, wheeze, sputum production, and shortness of breath can occur during the night and further disturb sleep (29, 30). In one study, 39% of patients experiencing nocturnal cough or wheeze reported difficulty initiating or maintaining sleep (24).

Evidence also suggests a possible bidirectional relationship between sleep quality and COPD-related clinical outcomes. Sleep disturbances may contribute to systemic inflammation, impaired immune function, reduced physical activity, cognitive changes, and poorer medication adherence, which in turn may worsen clinical outcomes and nocturnal COPD symptoms (31). Sleep disturbance in COPD has therefore been proposed to contribute to nonspecific daytime symptoms such as chronic fatigue, lethargy, and overall functional impairment (32).

Impaired sleep quality may further interfere with symptom relief over time. In a prospective patient cohort study, Lin et al. used minimum clinically important difference in CAT at 6-month from baseline as a symptom improvement indicator and showed that COPD patients with impaired sleep quality were less likely to achieve symptom improvement (33). In addition, patients with poor sleep quality were also at increased risk of future exacerbation compared to patients with good sleep quality. There was further an indication that patients with poor sleep had less effect of inhaled medication (33). Improving sleep quality in the COPD patient population may therefore be of importance to aid in other treatment in these patients.

## Sleep and psychological factors including quality of life in COPD

Sleep disturbances in COPD are closely linked with psychological symptoms and reduced quality of life, and patients with COPD may experience anxiety and depression alongside nocturnal respiratory symptoms such as dyspnea, coughing, sputum production, and hypoxemia (29, 30). These factors can interact with sleep problems, contributing to impaired daytime functioning and reduced overall quality of life (32). Chronic sleep disruption may also exacerbate fatigue and lethargy, further affecting daily activities and well-being (32).

## Sleep and pulmonary rehabilitation

Pulmonary rehabilitation (PR) is a key component of nonpharmacological management for COPD, and it has been shown not only to improve common symptoms such as dyspnea, reduce hospitalization rates, and enhance patients’ ability to perform activities of daily living (34) but also to reduce anxiety and depression among individuals with COPD (35). From a sleep perspective, PR shows improvements in subjective sleep quality; however, effects on objective measures of sleep such as sleep latency, total sleep time, sleep efficiency, and number of awakenings have not been shown (5, 35).

## Common sleep disorders in COPD; prevalence, risks, and treatment

As in the general population, the most common sleep disorders in COPD are insomnia, obstructive sleep apnea (OSA), and restless legs syndrome (RLS). Insomnia is usually defined as difficulty initiating sleep, difficulty maintaining sleep, and/or early morning awakening and is usually accompanied by a feeling of nonrestorative sleep. OSA is characterized by recurrent episodes of total (apnea) or partial (hypopnea) obstruction of the upper airway, due to muscle collapse in the pharynx during sleep and resulting in hypoxia, arousals (i.e. disrupted sleep), and snoring (36). RLS is a common sensorimotor disorder characterized by four diagnostic criteria: 1) a strong urge to move the legs, typically accompanied by uncomfortable or unpleasant sensations; 2) symptoms that begin or worsen during periods of rest or inactivity, such as sitting or lying down; 3) partial or complete relief of symptoms with movement, including activities such as walking or stretching, for as long as the movement continues; and 4) symptoms that are more pronounced in the evening or at night (37).

Insomnia has been reported in meta-analyzed data to be present in a third of patients with COPD although there are large variations depending on the cohort studied (38). As previously shown, patients or individuals with COPD indicate greater difficulty initiating and maintaining sleep as well as increased daytime sleepiness compared with the general population (11, 20). As in insomnia, in general, cognitive behavioral therapy for insomnia (CBT-I) has shown to give significant and long-term improvement in insomnia and improved sleep but clinically important improvement in fatigue and dyspnea (39).

Overlap syndrome, that is, having both COPD and OSA, has been reported ranging from approximately 10 to 66% (40, 41). However, in pooled data, in approximately one third of patients, an increasing prevalence over time has been reported (42). Prevalence nonetheless differs depending on populations, with possibly higher prevalence in Asian populations and in older populations (42). As both COPD and OSA are highly prevalent, it is not surprising that they may occur together. Although evidence does not suggest that patients with COPD are at increased risk of developing OSA (43), it does suggest that patients with overlap syndrome have worse quality of life and increased risk of developing cardiovascular diseases (44) compared with patients with COPD or OSA alone (43). Further, in patients with overlap syndrome, there are increased exacerbation and mortality rates compared to those with COPD but without OSA (43). Continuous positive airway pressure (CPAP) treatment for OSA has been shown to normalize the excessed exacerbation risk (45, 46).

RLS may impair sleep quality by prolonging sleep latency, increasing the frequency of nocturnal awakenings, and contributing to periodic limb movements during sleep. It has been reported to occur more frequently in patients with COPD than in the general population (47, 48) with a prevalence of approximately 21% (41). One proposed explanation for the higher prevalence of RLS in COPD is the presence of hypoxemia, which could play a role in the development or exacerbation of RLS symptoms (49). Treating RLS may improve both RLS symptoms (50) and improve sleep in COPD (41).

## Conclusions and future perspectives

Sleep in COPD is multifaceted (Figure 1), but although studies have assessed sleep in COPD, the area is still not extensively researched. Poor sleep quality is very common in the COPD patient population and is partly related to airway symptoms, psychological distress, and reduced quality of life. Studies on pulmonary rehabilitation and its effect on sleep are few, but there is an indication that rehabilitation improves sleep quality. Nonetheless, sleep is not routinely assessed and discussed in the clinic. In addition, there is likely an underdiagnosis of other sleep disorders such as OSA and RLS in the COPD patient population. Assessing sleep and sleep quality and treating both COPD and sleep disorders effectively in this patient population would likely improve not only sleep and airway symptoms but also quality of life.

**Figure 1:**
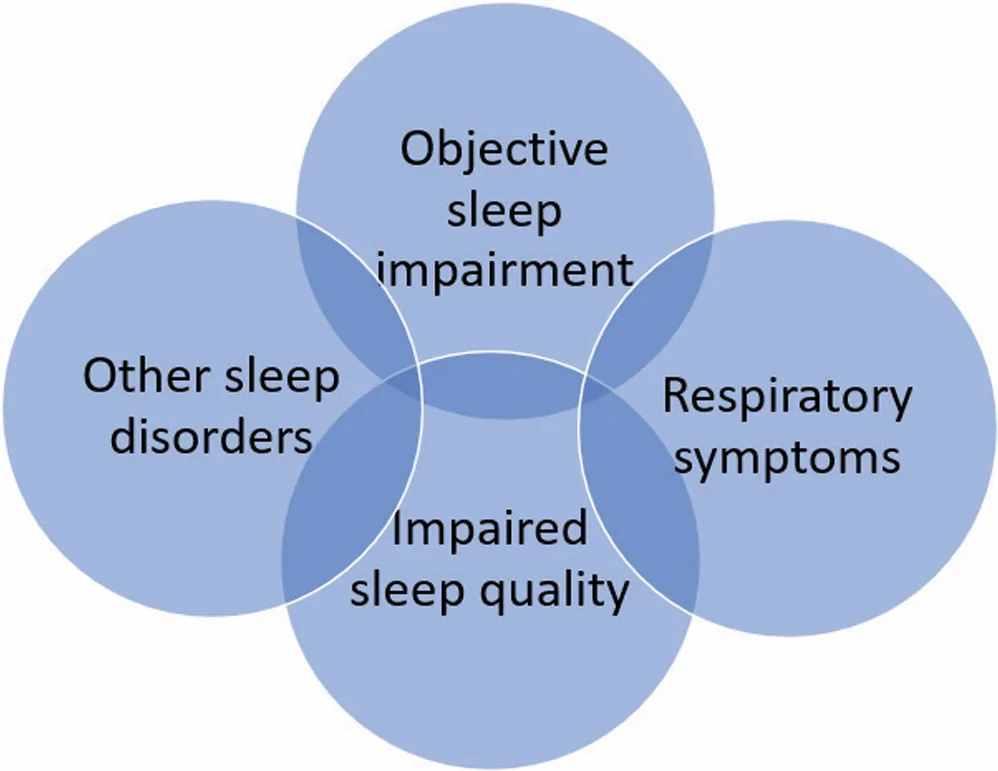
Schematic overview of the relationship between sleep and COPD.
